# A Biosurfactant-Sophorolipid Acts in Synergy with Antibiotics to Enhance Their Efficiency

**DOI:** 10.1155/2013/512495

**Published:** 2013-09-09

**Authors:** Kasturi Joshi-Navare, Asmita Prabhune

**Affiliations:** Biochemical Sciences Division, National Chemical Laboratory, Homi Bhabha Road, Pune Maharashtra 411008, India

## Abstract

Sophorolipids (SLs), biosurfactants with antimicrobial properties, have been tried to address the problem of antibiotic resistance. The synergistic action of SL and antibiotics was checked using standard microdilution and spread plate methods. With *Staphylococcus aureus*, SL-tetracycline combination achieved total inhibition before 4 h of exposure while tetracycline alone couldnot achieve total inhibition till the end of 6 h. The inhibition caused by exposure of bacterium to SL-tetracycline mixture was *~*25% more as compared to SL alone. In spite of known robustness of gram-negative bacteria, SL-cefaclor mixture proved to be efficient against *Escherichia coli* which showed *~*48% more inhibition within 2 h of exposure as compared to cefaclor alone. Scanning electron microscopy of the cells treated with mixture revealed bacterial cell membrane damage and pore formation. Moreover, SLs being a type of asymmetric bola, they are expected to form self-assemblies with unique functionality. This led to the speculation that SLs being amphiphilic in nature can span through the structurally alike cell membrane and facilitate the entry of drug molecules.

## 1. Introduction

The advent of antibiotics for treating bacterial infections is considered as one of the major advances in modern medicine [1]. Owing to the use and misuse of antimicrobials during past decades, the majority of clinically important bacteria have developed resistance to multiple antibiotics, thus reducing the utility of antibiotics. Such infections are severe, difficult to manage and require longer and more complex treatments [1–4]. 

In this scenario, it is imperative to discover fresh antimicrobials or new practices that are effective for the treatment of infectious diseases caused by drug-resistant microorganisms [3]. 

For the problem of antibiotic resistance different approaches such as nanotechnology, genomics are being tried out [1, 5]. These require detailed study for each drug and response by the target organism and are specific in nature. Combined antibiotic therapy is an alternative approach. In order to control multi-drug resistant pathogens such as tuberculosis and MRSA, that is, Methicillin resistant *Staphylococcus aureus*, clinicians have increasingly turned to multi antibiotic therapies [6]. The approach is being practised against Tuberculosis for over 50 years involving the drugs with different modes of action. Based on this approach, the drug synergism between antibiotics and bioactive plant extracts has been demonstrated [7].

Different biosurfactants possess antimicrobial property. Various biosurfactants, for example, from *Bacillus circulans, B. subtilis, B. licheniformis, Candida antarctica,* and *Pseudomonas aeruginosa, *have been reported to have potent antimicrobial activity. Some of them are effective against Multidrug resistant strains. By its structure, biosurfactant is supposed to exert its toxicity on the cell membrane permeability as a detergent-like effect [8]. Biosurfactants are coming up as emerging class of biomedical compounds. They are a suitable alternative to synthetic medicines and antimicrobial agents and could be used as safe and effective therapeutic agents or probiotics, especially at a time when drug resistance among causal organisms for many life-threatening diseases is on the rise [9]. Sophorolipid (SL) is a promising candidate for such applications being produced by nonpathogenic yeasts, such as *Candida bombicola, C. apicola,* and *C. bogoriensis*. They are generally present in the form of disaccharide sophoroses (2-O-*β*-D-glucopyranosyl-D-glucopyranose) linked *β* glycosidically to the hydroxyl group at the penultimate carbon of fatty acids [10]. Like other biosurfactants, SLs do possess antimicrobial property. The antimicrobial action is not merely restricted towards bacteria; they also act as antifungal, antialgal, antimycoplasma, and antiviral agents [11]. The proposed primary mechanism of action of these surfactants is membrane lipid order perturbation, which compromises the viability of microorganisms [12]. Moreover, SLs offer the advantages of biodegradability, low ecotoxicity, and the production based on renewable-resource substrates. The US FDA has also approved biosurfactants/sugar esters for the use in food and pharmaceuticals. SLs are not irritating to the skin, do not trigger allergic reactions, and have an oral safety level which is greater than or equal to 5 mL/kg weight. Cytotoxicity was evaluated with human epidermal keratinocytes and was proven to be low [13]. 

Sun et al., 2004, have demonstrated the synergistic effects of combination of SL and loess for harmful algal bloom mitigation to bring down the effective dose of both when used individually [14]. Mannosylerythritol lipid-A, a type of glycolipid biosurfactant containing cationic liposomes, promoted the gene transfection efficiency five to seven times with mammalian cultured cells [15]. Liposomes stand as promising candidates with wide applicability based on the drug delivery approach [16]. SLs bear 2 different polar heads on the 2 ends of the lipophilic core thus referred to as “asymmetric bolas.” Being amphiphilic in nature, they tend to form self-assemblies with unique structural and physiochemical properties as well as functionality [17]. Further upon modification, they can possibly be tuned into liposomes. Thus they have promising potential to be used as a drug delivery system. Moreover SLs possess a range of beneficial properties which make them suitable for pharmaceutical applications. No studies have evaluated the use of SLs in combination with antibiotics [18]. Therefore, here we have attempted to determine whether SLs can improve antibiotic efficiency. 

## 2. Materials and Methods

### 2.1. Microorganisms and Their Maintenance

- Nonpathogenic yeast, *Candida bombicola* (ATCC 22214), was used for the production of SLs. It was maintained on MGYP (malt extract—0.3 g%, glucose—2 g%, yeast extract—0.3 g%, peptone—0.5 g%, and agar—2.0 g%) slants. The microorganism was sub cultured in every 4 weeks and maintained at 4°C in a refrigerator.
- The test microorganisms, *E. coli* (ATCC-8739) and *S. aureus* (ATCC-29737), were procured from the National Collection of Industrial Microorganisms, NCL. The cultures were maintained on nutrient agar slants. The microorganisms were subcultured in every 4 weeks and maintained at 4°C in a refrigerator.

### 2.2. Chemicals and Reagents

All media, chemicals, and solvents used in this study were of analytical grade and supplied by either Hi-media pvt. Ltd., India, or Merck India ltd. 

The fatty acid precursor, Oleic acid, was purchased from Sigma Aldrich. The antibiotics, tetracycline HCl and cefaclor, were also purchased from Sigma and stored in refrigerator till required. Tetracycline is known to be soluble in water while cefaclor is partially soluble in water.

### 2.3. Synthesis and Extraction of SLs

For SL production, seed culture was prepared by inoculating 10 mL of fresh MGYP nutrient medium with *C. bombicola *ATCC 22214 followed by incubation at 30°C, 180 rpm for 24 h. This preinoculum was added to 90 mL MGYP nutrient medium in a 500 mL Erlenmeyer flask and incubated further for 48 h. Cells were harvested and washed twice with sterile distilled water. The cell pellets (biomass ~1.5 g dry weight in 100 mL medium) were redispersed in 100 mL of 10% glucose solution supplemented with 1 mL of Oleic acid (dispersed in 1 mL ethanol), and again incubation was continued for 96 h when a brown and viscous SL mass was seen settled at the bottom of the flask. It was separated using a pipette tip cut at nozzle and subjected to ethyl acetate extraction. Culture medium was centrifuged at 5,000 rpm, at 10°C for 20 min. The supernatant was extracted twice with equal volumes of ethyl acetate, the organic layer was dried over anhydrous Na_2_SO_4_, and the solvent was removed by rotary vacuum evaporation. The yellowish brown semicrystalline product was washed twice with n-hexane to remove unconverted fatty acid [19]. 

### 2.4. Characterization of SLs

#### 2.4.1. HPLC Analysis to Determine the Relative Proportion of Lactone and Acid Components

 The SL sample was subjected to HPLC analysis to get an idea about relative percentages of lactonic and acidic components of SLs based on standard sample run. The chromeline-Hitachi HPLC system was used along with C18 column (5 *μ*m, 150 × 4.6 mm). The solvent system used was MilliQ water-Acetonitrile (ACN). Total run time was 65 minutes. For the first 15 minutes, ACN was maintained at 20% and then it was gradually raised to 80% up to 40 minutes, further brought to 100% till 50 minutes, and thereafter maintained for 15 minutes. The run was performed at flow rate 0.5 mL/minute and 25°C. The compounds were detected by L-2490 UV detector at 207 nm. 

#### 2.4.2. Matrix Assisted Laser Desorption/Ionization Mass-Spectrometry (MALDI-MS) Study

SL sample 1 mg was dissolved in 1 mL of methanol. Further, 5 *μ*L of the sample was mixed with 20 *μ*L of dithranol matrix, and MALDI-MS study was done on AB SCIEX TOF/TOF 5800. 

### 2.5. Assay of Conjugative Action of SL and Antibiotic

For the assay of conjugative action of SL and antibiotic, cefaclor and tetracycline were used as antibiotics differing in their mode of action. Cefaclor inhibits the bacterial growth by preventing bacteria from forming the peptidoglycan cell walls [20]. Like beta lactam antibiotics, cefaclor targets the penicillin binding proteins or PBPs, a group of enzymes found anchored in the cell membrane which mediate the cross-linking of the cell wall, while tetracycline inhibits the protein synthesis by preventing the attachment of charged aminoacyl-tRNA to the ribosomal attachment (A) site [21]. Thus, tetracycline prevents introduction of new amino acids to the nascent peptide chain.

As can be seen from the modes of action, cefaclor molecules have to reach cell membrane while tetracycline molecules have to cross cell membrane and reach ribosomes to exert the inhibitory action. These 2 antibiotics were chosen in order to assess the role of SLs in enhancing the action of antibiotics differing in mode as well as site of action.

#### 2.5.1. Conjugative Effect of SL and Tetracycline against *S. aureus*

- Stock preparation: tetracycline stock was prepared by dissolving tetracycline HCl in sterile distilled water at the stock strength 1 mg/mL. SL stock was prepared in sterile distilled water by dissolving the appropriate amount of SL in sterile distilled water supplemented with 3% v/v alcohol. SL stock strength used was 10 mg/mL.
- Determination of minimum inhibitory concentrations of individual SL and tetracycline: in the first step, A_600_, that is, absorbance of bacterial suspension giving isolated colonies, was fixed and the same A_600_ was maintained throughout the experiment. Based on the prior experimentation, the SL concentration range was fixed for MIC determination as 100–600 *μ*g/mL while the concentration range used for tetracycline was 5–100 *μ*g/mL. Minimum inhibitory concentration (MIC) is defined as the lowest concentration of compound that inhibits visible growth of microorganisms on the culture plate. The concentration of SL or antibiotic at which no bacterial colony was observed on the plate was considered as minimum inhibitory concentration [22]. Based on the results of MIC determination experiments, the sublethal concentrations of both bioactive compounds were identified and used during the assay of conjugative effect.
- Time dependent assay of synergistic action along with controls: 4 test reactions were set up. The dilution scheme has been mentioned in Table 1. Cells without exposure to any bioactive agent served as the control.

During the sequential additions, SL and tetracycline were mixed thoroughly followed by addition of sterile distilled water and suspension. Thus the cells were exposed to the action of SL and tetracycline. Reaction mixtures were incubated at 28°C, 180 rpm for 6 h. The samples were removed at periodic intervals 2, 4, and 6 h and number of colony forming units (CFUs) were determined by spreading 50 *μ*L of mixture on nutrient agar plates. The plates were incubated at 28°C, and colonies were visualized after 24 h. All antibacterial activity tests were performed in triplicates, and average values were noted to certify the reproducibility. Colonies were counted and percentage cell survival was calculated using the following formula [23]:

% cell survival = no. of colonies on test plate ∗ 100/no. of colonies on control plate.

#### 2.5.2. Conjugative Effect of Cefaclor and SL against *E. coli*

Similar protocol as mentioned in the above experiment was followed. The SL stock was prepared at 10 mg/mL concentration, while cefaclor stock was prepared at 1 mg/mL concentration. The concentration range used for MIC determination of cefaclor against *E. coli* was 20–80 *μ*g/mL, while the concentration range used for SL was 100–1000 *μ*g/mL. 

Based on the results of MIC determination experiment, the sublethal concentrations of SL and cefaclor were decided and used for further experiment. 4 test reactions were set, namely, control, SL alone, SL with cefaclor, and cefaclor alone. Please refer to the Supplementary Information (available online at http://dx.doi.org/10.1155/2013/512495) for the dilution scheme. The sampling intervals, protocol, and data evaluation method were the same as mentioned above.

### 2.6. Scanning Electron Microscopy of Treated Cells

The cells were subjected to the action of SL and antibiotic combinations in respective proportions as **per** the protocol mentioned in Sections 2.5.1 and 2.5.2. After 4 h incubation with SL-antibiotic combination, the bacterial cell suspension was centrifuged. Cell pellet was resuspended in 200 *μ*L of sterile distilled water, and 10–15 *μ*L of it was drop-casted onto a silicon wafer for easier locating of bacterial cells and allowed to air-dry. Samples were sputter coated till a fine layer of 10 nm was formed (sputter coater; make—EMITECH, source- Au-Pd, gas-argon). The E-SEMs of the samples were then recorded at the resolution 3 nm at 30 kV under high vacuum (SEM; make—FEI, model-Quanta 200 #D Dual beam ESEM with EDAX, source—tungsten thermionic emission). The untreated healthy cells were also subjected to SEM as per the same procedure. They served as the control. 

## 3. Results

### 3.1. Characterization of SL

#### 3.1.1. HPLC Analysis to Determine Relative Proportions of Lactone and Acid Components

 As per the HPLC analysis, it was found that the SL sample contains around 75% of lactone form and remaining 25% of acidic form. The acidic SL forms get eluted first while the lactonic SLs, especially the acetylated ones, show longer retention times because of higher hydrophobicity [24]. Thus the peaks lying in the later half region were considered to be of different lactonic forms. (Refer to supplementary information for chromatogram.)

#### 3.1.2. MALDI-MS Study of SL

 Prominent peaks from the mass spectrum were correlated to sodium adducts [M^+^ + H^+^ + Na^+^] of the expected forms of SLs. Four different forms of Oleic acid derived SLs were detected. Diacetylated lactonic SL of Oleic acid, that is, 17-L-(-oxy)-octadecanoic acid 1,4′′-lactone 6′,6′′-diacetate, was detected with maximum % abundance. Diacetylated acidic form, monoacetylated lactonic SL, and monoacetylated acidic SL were also detected in relatively small proportions. Apart from Oleic acid derived SLs, different SL structures having Linoleic (C18:2), stearic (C18:0), and palmitic (C16:0) acids as the hydrophobic part were also detected. (Refer to supplementary information for mass spectrum.) The finding was in accordance with previous reports [25]. 

Dengle-Pulate et al., 2013 have synthesized SL using Lauryl alcohol as hydrophobic precursor and assessed its surface tension reducing property along with the SL derived from Oleic acid (SLOA) as a control. They have reported the Critical Micelle Concentration value of SLOA as 0.12 g/L and lowest surface tension as 34 mN/m [26].

#### 3.1.3. Conjugative Effect of SL and Tetracycline against *S. aureus*

**Figure 1:**
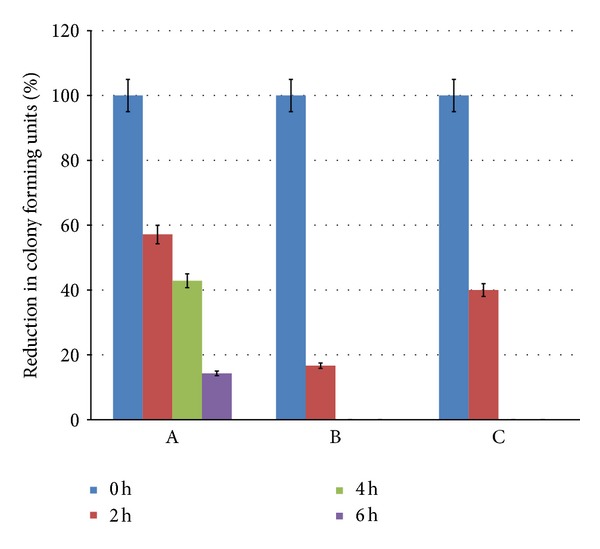
Synergistic action of tetracycline and SL against *S. aureus*. The graphs represent % reduction in colony forming units on exposure to different bioactive agents with respect to time 0, 2, 4 and 6 h. Bars represent A—effect of tetracycline alone, B—effect of tetracycline + SL, and C—effect of SL alone.

MIC of tetracycline against *S. aureus* (ATCC-29737) was found to be 150 *μ*g/mL, while that of SL was found to be 400 *μ*g/mL. Therefore it can be observed that both SL and tetracycline are capable of achieving inhibition. In order to rightly assess the time dependent trend of inhibitory effect of combination, sublethal concentrations of SL (300 *μ*g/mL) and tetracycline (15 *μ*g/mL) were exercised during the assay. The time dependent bacterial inhibition on the action of SL and tetracycline has been represented in Figure 1. It can be observed from Figure 1 that tetracycline alone cannot achieve total inhibition even after 6 h of exposure. SL alone was efficient against *S. aureus* at 300 *μ*g/mL and showed total inhibition within 4 h. However, it was worth noting that when both agents were used in combination, at 2 h exposure ~22% more inhibition was observed.

#### 3.1.4. Conjugative Effect of SL and Cefaclor against *E. coli*

**Figure 2:**
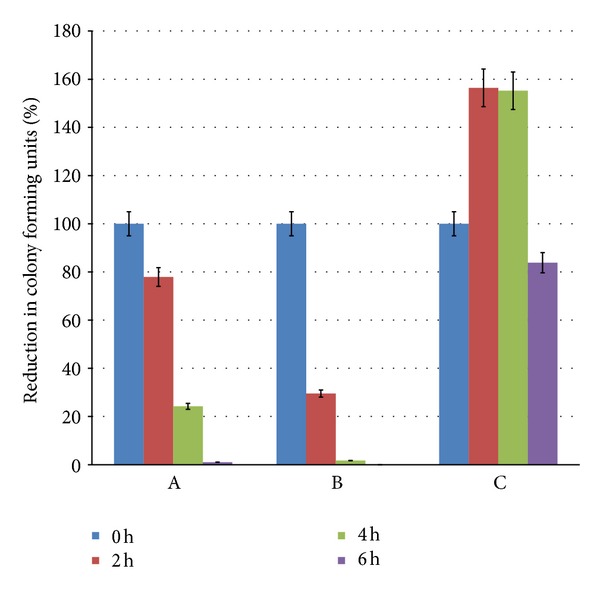
Synergistic action of cefaclor and SL against *E. coli*. The graphs represent % reduction in colony forming units on exposure to different bioactive agents with respect to time 0, 2, 4 and 6 h. Bars represent A—effect of cefaclor alone, B—effect of cefaclor + SL, and C—effect of SL alone.

MIC of cefaclor against *E. coli* (ATCC 8739) was found to be 200 *μ*g/mL, while SL alone was not inhibitory to *E. coli* till 1000 *μ*g/mL. For the assay of conjugative effect, sublethal concentrations of SL and cefaclor were decided to be 500 *μ*g/mL and 50 *μ*g/mL, respectively.  Figure 2 represents the comparative inhibitory action of cefaclor, SL, and their combination against *E. coli*. Cefaclor achieved almost total inhibition at the end of 6 h exposure. SL alone was unable to totally inhibit the bacterial growth, but when administered along with the antibiotic, it resulted in faster killing of the bacterium. It is worth to be noted that SL-cefaclor together could achieve ~98% killing within 4 h, while with cefaclor alone it required 6 h exposure to get equivalent effect. 

It has been observed through previous reports that SLs have better antibacterial action against gram-positive bacteria as compared to that against the gram-negative ones. Sleiman et. al., 2009, have checked the action of SLs and SL derivatives against different clinically relevant bacteria and reported that some activity was found against gram-positive bacteria, but against gram-negative bacteria considerably high concentrations showed trace activity [18]. Shah et al., 2007, also reported concurrent findings [27].

### 3.2. Scanning Electron Microscopy Images of the Bacterial Cells Treated with SL and Antibiotic

**Figure 3:**
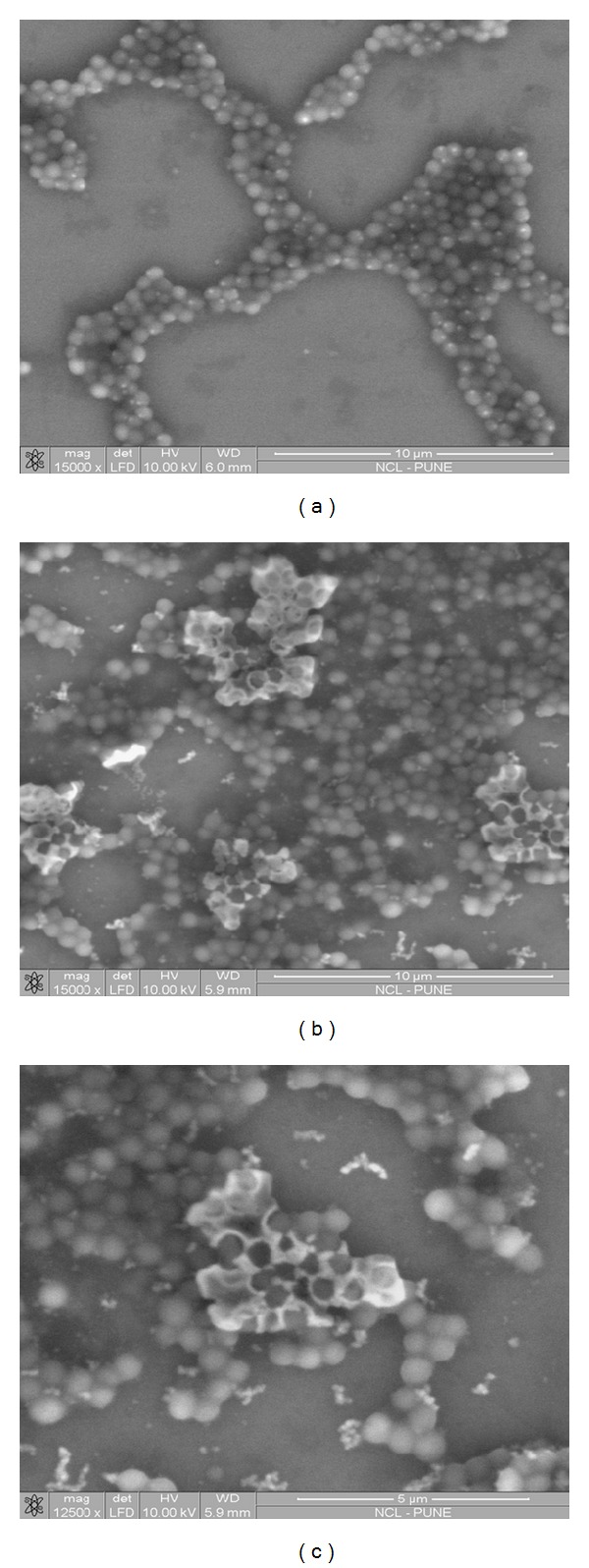
SEM images of (a) control—untreated *S. aureus *cells and (b) and (c) *S. aureus *cells treated with the mixture of SL and tetracycline. Cell membrane damage was evident from the images with accumulation of cell debris.

**Figure 4:**
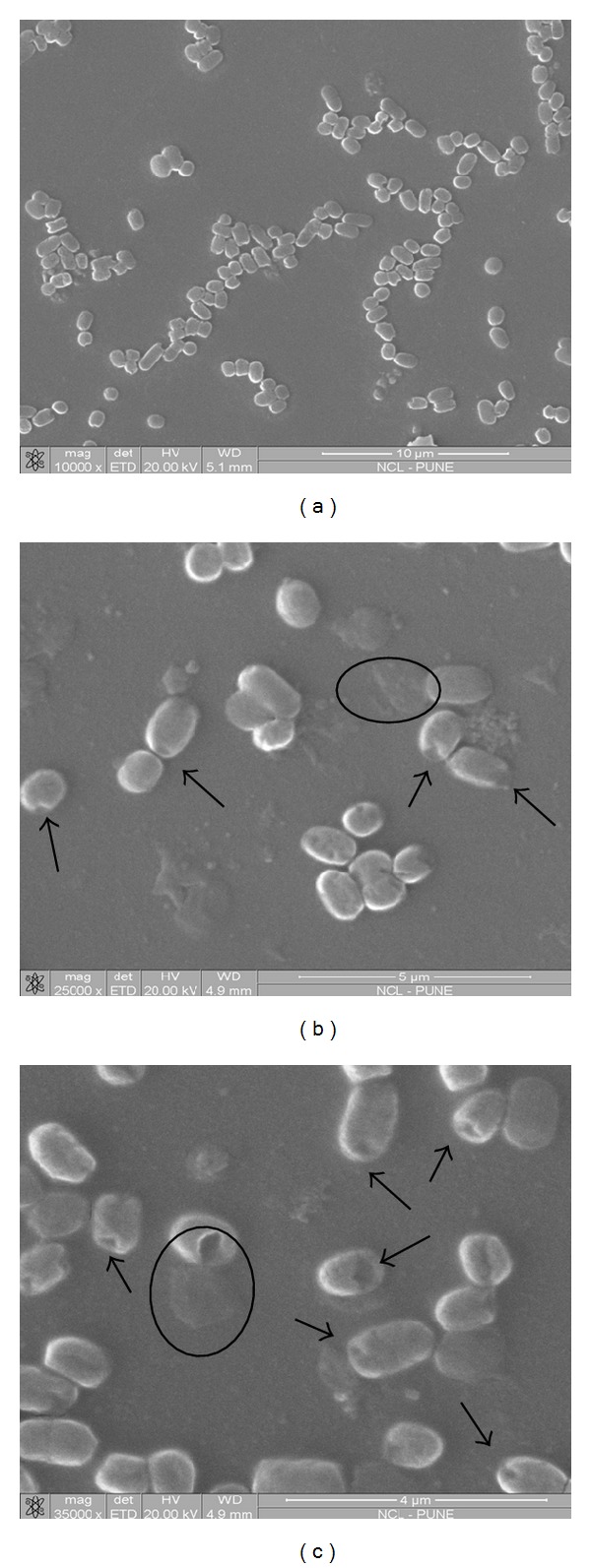
SEM images of (a) control—untreated *E. coli *cells and (b) and (c) *E. coli* cells treated with the mixture of SL and cefaclor. Disturbed cell integrity was evident from the images. Formation of pores (indicated by arrows) was visible with leakage of cytoplasmic contents.

Figure 3 shows the scanning electron micrographs of the *S. aureus* cells treated with the tetracycline-SL mixture, while Figure 4 shows the scanning electron micrographs of the *E. coli* cells treated with the cefaclor-SL mixture. Damage to cell membrane is evident from the images. The consequences of disturbed cell membrane integrity such as formation of membrane pores leading to leakage of cytoplasmic contents and accumulation of cell debris were also noted.

## 4. Discussion

Till date, no studies have evaluated the use of SLs in combination with antibiotics. Hence it is not known if these compounds might have antagonistic or synergistic effects when administered with antibiotics [18]. We have used the SL mixture during the study, knowing that natural synergism between SLs creates a better balance for many interfacial activities [28].

In this study, *S. aureus* and *E. coli* were used to evaluate the synergistic action of SLs with different antibiotics. These organisms are commonly occurring pathogens. *S. aureus* is a gram-positive coccus, currently responsible for the majority of skin and soft tissue infections (SSTIs) as per survey report carried out in the United States. This bacterium is commonly found asymptomatically in healthy individuals, colonizing the anterior nares and other sites of the body, such as the skin and gastrointestinal tract. However, *S*. *aureus* can be extraordinarily pathogenic, causing a broad range of morbid states from serious skin infections, such as cellulitis and abscesses, to endocarditis and sepsis. *S. aureus* is rapidly evolving resistance to contemporary topical as well as systemic antibiotics [29]. Another index bacterium used in the present study, *E*. *coli* is found in the lower intestine of warm blooded animals. *E*. *coli* is more than just a harmless intestinal inhabitant; it can also be a highly versatile pathogen that can be frequently deadly. Several different *E*. *coli* strains cause diverse intestinal and extraintestinal diseases by means of virulence factors that affect a wide range of cellular processes [30]. To include the bacterium here was important so as to observe the antimicrobial effect of cefaclor and cefaclor in combination with SL on the gram-negative bacteria that have a thin peptidoglycan layer adjacent to the inner cytoplasmic membrane, which makes them have little resistance against cefaclor. 

**Figure 5:**
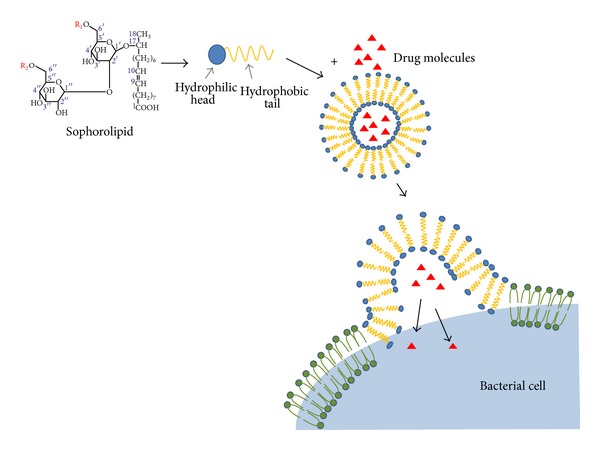
Schematic diagram displaying the proposed mechanism of SL mediated drug entry facilitation across cell membrane.

The scanning electron micrographs exhibit a range of morphological changes suggestive of damage to cell membrane, and the consequences of disturbed cell membrane integrity such as formation of membrane pores leading to leakage of cytoplasmic contents and accumulation of cell debris were noted. It should be noted that though the antibiotics differ in their action, similar features of cellular damage were observed in both cases. So it can be said that the inhibitory action involved cell membrane lipid order perturbation in addition to the action of antibiotic. The inhibitory effect of antibiotics was observed at lower concentrations when coadministered with SLs. Probably SLs have enhanced the drug action by facilitating the entry across cell membrane thus achieving requisite intracellular antibiotic concentration at low dosage. A hypothesis has been proposed about the entry of antibiotic molecules in presence of SL as schematically represented in Figure 5.

*Hypothesis about Mechanism of Drug Entry Facilitation by SLs.* Naturally evolution does not provide any active transporter for antibiotics, and a passive diffusion process facilitated by channels must be invoked [31]. Antibiotic agents are thought to diffuse freely through the cell wall of gram-positive bacteria. However, in gram-negative bacteria the diffusion of a given antibiotic agent depends on the permeability of the outer membrane. This permeability is determined by the particular structure of the membrane, which is composed of proteins and an asymmetric lipid bilayer [32]. The outer membrane of bacteria contains various protein channels, called porins, which are involved in the influx of various compounds, including several classes of antibiotics. Bacterial adaptation to reduce influx through porins is an increasing problem worldwide that contributes, together with efflux systems, to the emergence and dissemination of antibiotic resistance. Gram-negative bacteria are responsible for a large proportion of antibiotic-resistant bacterial diseases. These bacteria have a complex cell envelope that comprises an outer membrane and an inner membrane that delimit the periplasm [33]. Tetracycline resistance is often due to the acquisition of new genes, which code for energy dependent efflux of tetracyclines [21]. Therefore while addressing the issue of antibiotic resistance, enhancing the permeability of drugs is of fundamental importance. 

The SLs, as described earlier, are capable of forming micelles, bilayer structures, and self-assemblies which can enclose the water soluble drugs. When administered together, SLs can span through the structurally alike cell membrane lipid bilayer and deliver the drug molecules to the cell interior. Structural studies of SL-antibiotic mixture with sophisticated instrumentation may throw some light on interaction of the two such as micellarization. SLs are known to have better antibacterial action against gram-positive bacteria, while large doses of the SLs are required to demonstrate any antibacterial activity, especially with Gram-negative bacteria [15, 27]. In agreement with this fact, SL alone could not inhibit the growth of *E. coli*. But in case of cells treated with the combination of SL and cefaclor, total inhibition was achieved much faster as compared to the sample treated with cefaclor alone. Therefore it can be concluded that the enhanced inhibitory effect is not due to additive action of two antimicrobial agents. Hence the enhanced efficiency of cefaclor-SL combination against *E. coli* can be considered as a proof for the argument that better performance is due to facilitation of entry of drug molecules by SLs.

Lactonic SLs have been reported to have better surface tension lowering and antimicrobial activity as compared to the acidic form [11]. Therefore we can expect that increasing the percentage of lactonic SL will improve the antibacterial property of SL.

Lactonic SLs are more hydrophobic as compared to the acidic SL. Packing constraints are associated with the lactonic SLs due to closed-ring structure of alkyl chain. Self-assembled structures of lactonic SLs can be unilamellar vesicles or tubules, whereas acidic SLs generally form small globular micelles. Therefore pertaining to the hypothesis we have speculated about the entry of drug molecules, it can be said that varying the lactone: acid percentage will also affect the affinity of micellar structures towards water soluble and water insoluble drugs [17].

Combined antibiotic therapy has been shown to delay the emergence of bacterial resistance and also produce desirable synergistic effects in the treatment of bacterial infections [7]. In case of nanoparticles, when they are used together with antibiotics, advantage is conferred that if bacteria have resistance against one of the components, a further component could kill them in a different manner [22]. Similarly, SLs being antimicrobial in nature, when coadministered with antibiotics, reduce the likelihood of bacterial survival as well as development of resistance. Also because of enhanced entry of antibiotic molecules, the desired inhibitory effect may be achieved at low concentration of antibiotic.

The structure of the surfactant dictates the HLB, that is, hydrophilic lipophilic balance value of the molecule. This value indicates whether the molecule will form water in oil, that is, W/O emulsions, or oil in water, that is, O/W emulsions. Depending on the solubility of the antibiotic, choice of surfactant can be made. SLs are known as “tailor-made molecules.” It is possible to achieve SL structural variation by varying the hydrophobic or hydrophilic carbon substrates fed during the synthesis procedure. 

Therefore due to enhanced entry and simultaneous action of 2 inhibitory agents, it can be expected that the coadministration of suitable SL and antibiotic will handle the infection efficiently. SLs can be safely administered till considerably high dosage, that is, 5 mL/kg of body weight, which is another fact favouring its pharmaceutical usage [13]. 

## 5. Conclusion

Coadministration of SL enhanced the action of antibiotics in representative gram-positive and gram-negative bacteria. These bacteria differ in their cell membrane structures and also the machinery to prevent the entry of antibiotic molecules. Considering the ability of SLs to form self-assemblies it has been speculated that self-assembled SLs can span through the structurally alike bacterial cell membrane and thus facilitate the entry of drug molecules. Self-assembled system may enhance solubility of antibiotic or offer shielding from the environment hence resulting in improved efficiency. Additionally SLs are antimicrobial in nature. Hence, if co-administered with another inhibitory agent, the mixture reduces the likelihood of bacterial survival and probably the development of resistance as the bacteria have to combat against 2 agents.

## Supplementary Material

The supplementary information contains (a) Dilution scheme for the assay of conjugative action of Sophorolipid and cephaclor against E. coli. The dilution scheme is similar to the one represented in Table 1. (b) HPLC chromatogram of the crude sophorolipid preparation which puts some light on the relative lactone: acid percent composition of the crude sophorolipid. (c) MALDI/MS spectrum of the sophorolipid preparation in which the peaks corresponding to prominent forms of oleic acid derived sophorolipid have been marked with arrows. Along with oleic acid derived sophorolipid, few more SL forms containing other fatty acids as hydrophobic tail have been listed in a table.

## Figures and Tables

**Table 1:** Dilution scheme used for the assay of conjugative action of SL and tetracycline against *S. aureus. *

Sr. no.	Test reaction description	Volume of SL stock (*μ*L)	Volume of antibiotic stock (*μ*L)	Volume of sterile distilled water (*μ*L)	Volume of bacterial suspension (*μ*L)	Total volume (*μ*L)
1	Control	—	—	800	200	1000
2	SL alone	30	—	770	200	1000
3	SL + tetracycline	30	15	755	200	1000
4	Tetracycline	—	15	785	200	1000
