# Supplementary material for: A Biosurfactant-Sophorolipid Acts in Synergy with Antibiotics to Enhance Their Efficiency
Source: Biomed Res Int. 2013 Sep 9;2013:512495. doi: 10.1155/2013/512495 (PMC3782141; doi:10.1155/2013/512495)
Supplement: Supplementary file 1 — The supplementary information contains (a) Dilution scheme for the assay of conjugative action of Sophorolipid and cephaclor against E. coli. The dilution scheme is similar to the one represented in Table 1. (b) HPLC chromatogram of the crude sophorolipid preparation which puts some light on the relative lactone: acid percent composition of the crude sophorolipid. (c) MALDI/MS spectrum of the sophorolipid preparation in which the peaks corresponding to prominent forms of oleic acid derived sophorolipid have been marked with arrows. Along with oleic acid derived sophorolipid, few more SL forms containing other fatty acids as hydrophobic tail have been listed in a table. [file 512495.f1.docx]

Supplementary information-

1. Table legend: Dilution scheme used for the assay of conjugative action of SL and Cefaclor against *E. coli*

| Sr. no. | Test reaction description | Volume of SL stock (µl) | Volume of antibiotic stock (µl) | Volume of sterile distilled water (µl) | Volume of bacterial suspension (µl) | Total volume  (µl) |
| --- | --- | --- | --- | --- | --- | --- |
| 1 | Control | - | - | 800 | 200 | 1000 |
| 2 | SL alone | 50 | - | 750 | 200 | 1000 |
| 3 | SL + Cefaclor | 50 | 50 | 700 | 200 | 1000 |
| 4 | Cefaclor | - | 50 | 750 | 200 | 1000 |

1. HPLC chromatogram of the crude sophorolipid preparation

Figure legend: HPLC pattern of the SL preparation

1. MALDI/MS spectrum of the sophorolipid preparation


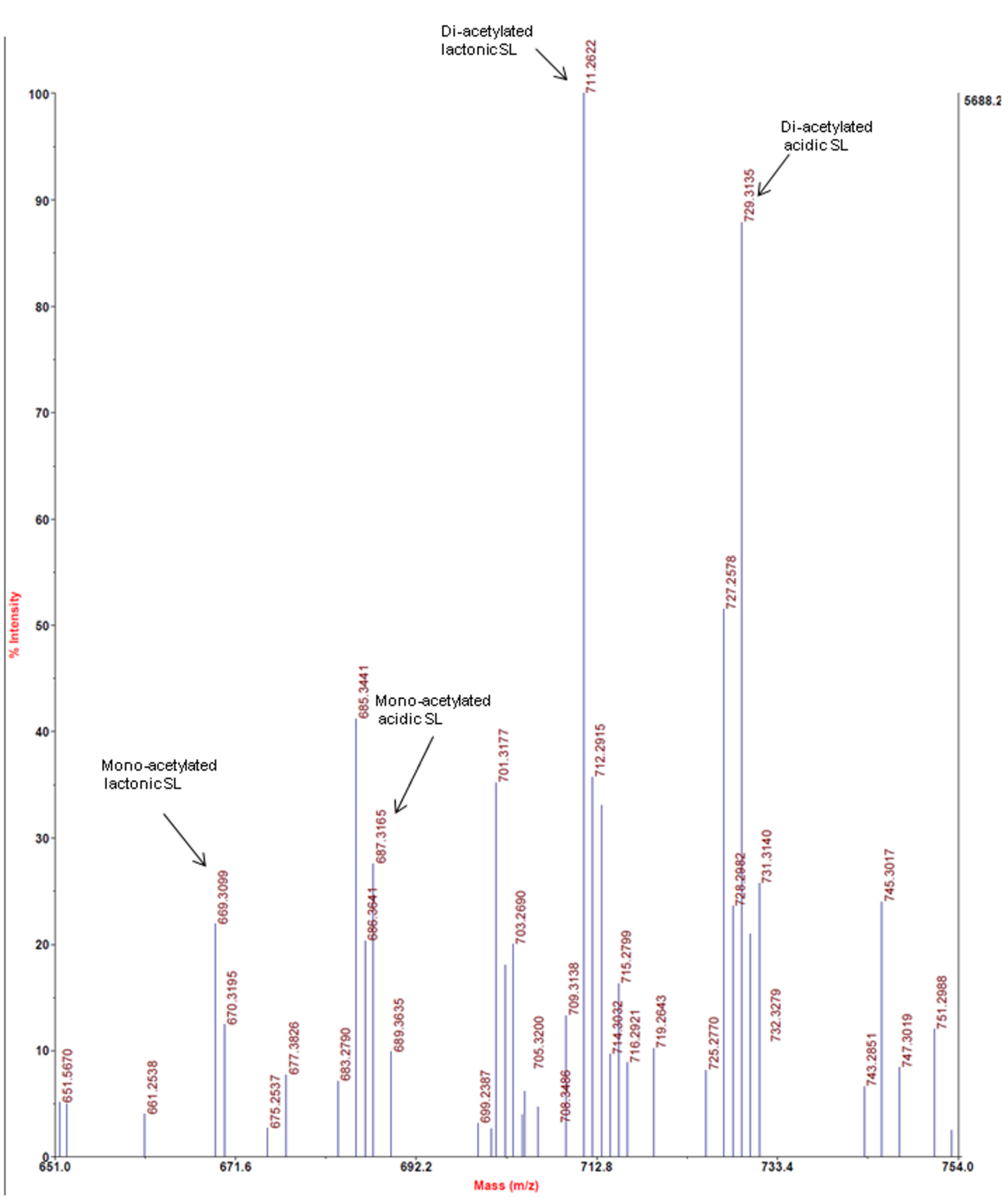


Figure legend- MALDI-MS data of SL preparation. [M^+^+H^+^+Na^+^] i.e. sodium adducts of the different structural forms of Oleic acid derived SLs have been indicated.

**Peaks corresponding to SLs having fatty acids other than Oleic acid**

| SL structure | m/z | M^+^+H^+^+Na^+^ |
| --- | --- | --- |
| Di-acetylated SL of C18:2 Acidic form | 703 | 727 |
| Di-acetylated SL of C18:0 Acidic form | 707 | 731 |
| Di-acetylated SL of C18:2 Lactonic form | 685 | 709 |
| Di-acetylated SL of C16:0 Lactonic form | 661 | 685 |
